# Similar responses to EQ-5D-3L by two elicitation methods: visual analogue scale and time trade-off

**DOI:** 10.1186/s12874-020-01008-9

**Published:** 2020-05-14

**Authors:** Xiuying Wang, Lin Zhuo, Yifei Ma, Ting Cai, Aviva Must, Ling Xu, Lang Zhuo

**Affiliations:** 1 Department of Nephrology, Xuzhou Central Hospital, Xuzhou Clinical School of Xuzhou Medical University, Xuzhou, Jiangsu China; 2 School of Basic Medical Sciences, Xinxiang Medical University, Xinxiang, Henan China; 3 School of Public Health, Xuzhou Medical University, Xuzhou, 221004 Jiangsu China; 4 Department of Public Health and Community Medicine, Tufts University School of Medicine, Boston, MA USA; 5 Center for Health Statistics and Information, National Health Commission, Beijing, China

**Keywords:** Quality of life measurement, EQ-5D-3L, Visual analogue scale (VAS), Time trade-off (TTO), Valuation method

## Abstract

**Background:**

Health-related quality of life (HRQoL) is often measured using EQ-5D-3L by the elicitation methods of visual analogue scale (VAS) and time trade-off (TTO). Although many countries have constructed both national VAS and TTO value sets, the fact that VAS and TTO value sets produces different values bewilders researchers and policymakers. The aim of this study is to explore certain conditions which could yield similar value sets using VAS and TTO.

**Methods:**

A homogeneous sample of medical school students was selected to value 18 hypothetical health states using VAS and TTO methods. The 18 hypothetical health states were produced by orthogonal design (L18, 2*3^7). The range of rescaled values was transformed into − 1 ~ 0 ~ 1. The investigations via different methods were carried out by computer-assisted personal interviewing with a wash-time interval of 72 h. Value sets for VAS and TTO were constructed using general least square regression models. Independent variables were composed of 10 dummy variables from 5 dimensions and including or omitting both constant and N3 terms.

**Results:**

Three hundred thirteen medical students participated. The mean age was 21.03 ± 0.44 years and 56.2% were female. The four regression models (for each method with and without constant and N3 terms) were all statistically significant (*P* < 0.05) with high goodness-of-fit (Adj. *R*^*2*^ > 0.94 and MAE < 0.033). Differences between the coefficients of the 10 dummy variables corresponding to each model were all less than 0.059. Pearson correlation coefficients between observed means and predicted values exceeded 0.981. Fitted curves of VAS and TTO largely coincided.

**Conclusions:**

VAS and TTO can generate similar responses under certain conditions, suggesting that the two valuation methods could be equivalent intrinsically. The VAS method appears a more valid approach for valuation in the general population due to its greater simplicity and feasibility.

## Background

Worldwide, people are getting healthier, living longer and spending more time with often debilitating chronic diseases [1]. Patients with chronic diseases are confronted with reduced quality of life (QoL) while the management of chronic disease significantly drains the human and financial resources in the health system [2]. The past decades have seen a growing body of research into quality-adjusted life-years (QALYs) which have emerged as one of the key outcome measures in health resources allocation [3, 4]. QALYs are calculated using the time in a specific health state multiplied by a score representing the value of that specific health state [5]. Health-related quality of life (HRQoL) has been widely used to indicate the utility of any specific health state [6]; the score of utility is anchored at 0 (death) and full health (1). To date, several multi-attribute utility-based instruments (MAUI) have been proposed for measuring HRQoL, e.g. EQ-5D [7], Short Form 36 (SF-36) [8], WHOQOL-BREF [9], and Health Utility Index (HUI) [10, 11]. Among these, EQ-5D is the most concise [12, 13].

The EQ-5D, an acronym for “European Quality of Life with 5 Dimensions”, is a generic instrument that was published by the EuroQol Group in 1990 [14], consisting of a EQ-5D descriptive system and a Visual Analogue Scale [3]. The EQ-5D descriptive system comprises 5 dimensions (mobility, self-care, usual activities, pain/discomfort, and anxiety/depression) with 3 levels each (no problem, moderate problem, and severe problem), thus defining 243 (3^5^) distinct health states to characterize HRQoL. EQ-5D has been used in health-value research [15], cost-utility analysis [16], and population health services surveys [17, 18] in many countries.

Visual analogue scale (VAS) or time trade-off (TTO) [19] are commonly used for EQ-5D value set elicitation. The first pair of VAS and TTO value sets for the EQ-5D was derived from the general population of the United Kingdom in 1990s [20]. Subsequently, Germany, Spain, Denmark, Argentina, and Sweden etc. have generated both VAS and TTO value sets [20, 21]. However, all value sets identified in pairs offered inconsistent values for HRQoL, that is, the VAS and TTO value sets in the same country generated different values for same health states [20, 21]. For example, the VAS value set predicts lower scores than TTO value set for mild health states and higher scores for severe health states in the United Kingdom [21]; in Sweden, the predicted TTO values are uniformly higher than VAS values [22]. Dominant explanations for the discrepancies include: that different instruments measured different aspects of health-related quality of life and thus yielded different results [23]; VAS values did not relate to years of life, VAS values were not useful for economic analysis [24]. However, some health economic critics contended that the discrepancies in the value sets preclude reaching a valid conclusion [25]. Furthermore, some policymakers argued that these inconsistencies undermine the fundamental strength and validity of HRQoL measurement [4, 26]. These criticisms demonstrate the need for better understandings of these discrepancies.

There have been several investigations in recent decades into the causes of the observed disagreement [27, 28]. In 2009, Craig et al. demonstrated the extent of agreement between VAS and ranking, another elicitation method of health states [29, 30]. To date, there has been relatively little research focusing on the agreement between VAS and TTO. This study attempts to redress this deficit. We assert that the difference between the two elicitation methods stem from several biases. First, the contrasting levels of complexity between the two methods may result in differences in comprehension and adherence of the respondents to the protocol [31]. Second, the lack of a lower boundary in the transformed HRQoL values gives respondents too much “free space” in which to make a choice [32]. Furthermore, traditional interviews, with pencil and paper, cannot provide immediate feedback on inconsistencies, which inevitably results in errors and decreases validity. Finally, pre-selected health states in MVH protocol comprised an empirical sample, which does not represent the full underlying population of all health states. By eliminating these potential sources of biases, we hypothesized that we would achieve relatively similar responses using VAS and TTO methods. If achieved, it would serve to strengthen the foundation of HRQoL measurement.

## Methods

The current experimental study was designed to obtain value sets by VAS and TTO elicitation methods using an adapted measurement and valuation of health (MVH) protocol. We improved our study design in several aspects as outlined below.

### Homogeneous sampling

Mortimer et al. have argued that variation between individuals is more important in explaining variation in predicted quality-of-life weights than the choice of elicitation technique [33]. In comparison to VAS, TTO is cognitively burdensome and challenging to administer, as pointed out by Craig [24]. These observations suggest that sample homogeneity offers the opportunity to improve both validity and feasibility. Therefore, we chose a homogeneous sample of third-year undergraduates in Xuzhou Medical University.

### Sample size

According to Chevalier et al. [34], the sample size needed for a general value set was calculated following the formula: $\mathrm{n}\kern0.5em =\kern0.5em \frac{{\mathrm{Z}}_{1-\frac{a}{2}}^2}{\delta^2}{\sigma}^2$, where ${\mathrm{Z}}_{1-\frac{\alpha }{2}}$ represents the percentile of the normal distribution used as the critical value in a two-tailed test of size α (${\mathrm{Z}}_{1-\frac{\alpha }{2}}$ =1.96 for a 0.05 level test). δ is the tolerated margin of error (δ =0.05), and σ is an estimate of the standard deviation from a pilot survey (σ = 0.4). Application of this formula results in 246 samples needed to obtain an estimation of the mean with a 95% (1-α) probability that the true mean falls in the interval of observed mean ± δ. Taking into account the expected compliance rate, we increased the sample size to 350, which is accordant to the one recommended by Lamers et al. [35].

### Selection of health states

Although 43 health states in MVH protocol [36] and 97 health states in Paris protocol [37] were recommended to derive the EQ-5D value set, these states were chosen arbitrarily. A fully balanced set of health states is needed to represent all health states. Put another way, a representative sample of health states is critical to draw a valid inference. In this study, a total of 18 hypothetical health states was created by orthogonal design (L18, 2*3^7), which is an approach increasingly adopted [38, 39]. Table 1 presents the 18 hypothetical health states used in this study, ordered as in the actual protocol.

**Table 1:** 18 health states created by orthogonal design (L18,2*3^7)

State number	Mobility	Self-Care	Usual Activities	Pain/Discomfort	Anxiety/Depression
1	1	1	1	1	1
2	3	1	1	2	2
3	2	2	1	1	3
4	1	3	2	1	2
5	1	2	3	2	1
6	2	1	2	3	1
7	2	2	2	2	2
8	2	3	3	1	1
9	1	1	3	3	2
10	3	2	1	3	1
11	3	1	2	1	3
12	1	3	1	2	3
13	1	2	2	3	3
14	3	3	2	2	1
15	2	1	3	2	3
16	2	3	1	3	2
17	3	2	3	1	2
18	3	3	3	3	3

The numbers 1, 2, and 3 in five dimensions represent level 1 (no problem), level 2 (moderate problem) and level 3 (severe problem), respectively

### The valuation tasks

Because of its greater simplicity, VAS valuation was conducted prior to TTO valuation. According to Ebbinghaus’ theory of forgetting curve [40], a 72-h between-task interval was employed to reduce the negative influence of the retention. Before each experimental trial, the participants attended a classroom instruction 1) explaining the purpose of the study; 2) introducing EQ-5D and VAS or TTO valuations; 3) demonstrating the interface of computer-assisted personal-interviewing (CAPI) software and its functions; and 4) reinforcing with opportunities for practice with the CAPI software. In the software, the state of full health (11111) was assigned a value of 10 as an anchor point. The participants were required to value the other 17 health states using VAS or TTO methods.

In the process of VAS valuation, a tailored scale, similar to a thermometer, was shown to the participants on the CAPI interface. “-10 (worst imaginable state)” was labeled at the lower end of the scale; “0 (dead)” was labeled at the midpoint of the scale; “10 (full health)” was labeled at the top of the scale. Participants were then asked to rank one health state each time on the scale at the point x, to indicate how good or bad they deemed the state. A utility weight for each state was calculated as x/10. Accordingly, the range of the transformed values was − 1 ~ 0 ~ 1. In this scheme, − 1, 0, and 1 indicate the imaginable worst state, being dead, and full health, respectively [32, 41].

In the process of TTO valuation, the participants were assumed to be in the impaired health state for 10 years followed by immediate death. If the health state was considered better than death (BTD), then the participants were asked to trade for t years in full health, where t decreased from 10 to 0 with a decrement of 1 year followed by immediate death. The elicitation process ended when the participant was indifferent to either in full health for t years or in the impaired state for 10 years. One decimal was permitted if the participant believed that 1 year was not adequately precise. Utility weight for BTD was calculated as t/10. In the case of a health state being regarded as worse than death (WTD), the participants would prefer to live t years in full health to compensate for tolerating the impaired state for (10-t) years. The years for compensation decreased from 10 to 0 with a decrement of 1 year followed by immediate death. The elicitation process ended when the participant was indifferent to either (10-t) years of tolerating the health state plus t years of full health or immediate death. A decimal was permitted if the participant believed 1 year was not adequately precise. Utility weights for WTD states were calculated as -t/10. The range of transformed TTO values was also − 1 ~ 0 ~ 1. Thus, the two evaluation methods are on the same scale, similar to the EuroQol Group Valuation Technology (EQ-VT) protocol described by Oppe M. et al. [42].

### Quality control

The valuation processes were carried out in a computer lab. After instruction, the participants carried out the task individually. There were three types of approaches to optimize the quality of valuation. These are: an acceptable predicted value for the distinct participant; the number of inconsistencies is three or less; the absolute value of the difference between the standard deviation of the 18 health states and 2.5 is less than 0.5. The three types of approaches are explained in detail as below.

After valuing 18 hypothetical states, participants were asked to describe their own health state using the EQ-5D-3L descriptive system in the CAPI software. Then a predicted value of the participant was derived from a multiple linear regression model, which was generated from the previously valued 18 health states. Additionally, the number of logical inconsistencies was also fed back to the participant based on the multiple linear regression models. For example, when disutility is adopted as independent, the level 3 (severe problems) of each dimension should have a higher value (in absolute term) than the level 2 (moderate problems), and the level 2 should be higher than the level 1. Accordingly, there are 15 comparable pairs in total. The standard deviation of the 18 values of the hypothetical states was also presented. Participants were asked to optimize their previous values to improve the results, but they retained the ability to keep the initial results if they wanted.

Because individuals differ in their ability to grasp the abstract health states, we used three distinct approaches to accommodate individual differences: including numbers, words, and pictures. For example, the abbreviation 31122 represents a health state with extreme problems in walking, no problems with self-care, no problems with performing usual activities, moderate pain, and moderate anxiety. The numbers and words were both presented. Additionally, a cobweb diagram [43] illustrating the hypothetical state on the five dimensions and the three severity levels was also shown to the respondents on the interface of the CAPI. (The interfaces of the CAPI software are presented in the “Supplementary Materials”.)

### Data cleaning

Standard protocols for data cleaning do not yet exist. We excluded the participants who met at least one of the following criteria: 1) could not complete the value task; 2) had more than four logical inconsistencies; 3) produced more than two outliers. Outliers were defined as those meeting all the following 1) data-points distinctly isolated from the whiskers in a box-plot; 2) distance between outliers and the nearest quartile was more than 3 times the interquartile range; and 3) values less than the 5th percentile or more than 95th percentile [43].

### Modeling

All 18 health states, including the anchor point 11111, were all used for building models for maximum use of the data. Studies have shown that the anchor point exerts a great impact on the value set [44, 45].

We employed disutility (1-utility) as the dependent variable. Independent variables included 10 dummy variables and N3. The dummy variables consisted of MO2, MO3, SC2, SC3, UA2, UA3, PD2, PD3, AD2, and AD3, which represent the main effect of any movement from no problem to moderate or severe problem for each dimension. N3 equals 1 if any dimension is level 3, 0 otherwise [43]. Table 2 shows the dummy variables used in the modeling.

**Table 2:** Definition of dummy variables and model specification

Dummy variables	Definition	
MO2	1 if mobility is level 2; 0 otherwise	
SC2	1 if self-care is level 2; 0 otherwise	
UA2	1 if usual activities is level 2; 0 otherwise	
PD2	1 if pain/discomfort is level 2; 0 otherwise	
AD2	1 if anxiety/depression is level 2; 0 otherwise	
MO3	1 if mobility is level 3; 0 otherwise	
SC3	1 if self-care is level 3; 0 otherwise	
UA3	1 if usual activities is level 3; 0 otherwise	
PD3	1 if pain/discomfort is level 3; 0 otherwise	
AD3	1 if anxiety/depression is level 3; 0 otherwise	
N3	1 if at least one level 3; 0 otherwise	
constant	loss of utility of any health state away from full health	
**Model Specification**	***f*(x)**	**Methods**
Model 1	*f* (MO2 MO3 SC2 SC3 UA2 UA3 PD2 PD3 AD2 AD3 N3)	VAS model with constant and N3
Model 2	*f* (MO2 MO3 SC2 SC3 UA2 UA3 PD2 PD3 AD2 AD3 N3)	TTO model with constant and N3
Model 3	*f* (MO2 MO3 SC2 SC3 UA2 UA3 PD2 PD3 AD2 AD3)	VAS model without constant and N3
Model 4	*f* (MO2 MO3 SC2 SC3 UA2 UA3 PD2 PD3 AD2 AD3)	TTO model without constant and N3

We adopted a general least squares (GLS) regression method to produce multilevel models, since each participant valued 17 health states. Specifications of the models defined in this study are also presented in Table 2. All statistical analysis and modeling were conducted using STATA/SE 12.0 (StataCorp, College Station, TX) with α set at 0.05 to declare statistical significance.

## Results

### Sample characteristics

We enrolled 350 medical students in this survey and excluded 37 participants including 2 for incompleteness, 26 who had more than 4 inconsistencies, and 9 who had more than 2 outliers. The final sample consisted of 313 participants with an average age of 21.03 ± 0.44 years; 56.2% were female.

### Descriptive statistics of 18 health states

Overall, 5634 VAS values (96.1% BTD) were collected with an average of 4.788 ± 2.703; the state of 33,333 received 197 out of 218 WTD values. All 5634 TTO values (94.2% BTD) were collected with an average of 4.310 ± 2.457; the state of 33,333 received 260 out of 326 WTD values. Other health states had only a small number of WTD values (VAS < 3, TTO < 6).

Raw data were then transformed into utility values by dividing by 10. Table 3 shows the mean, standard deviation, median, and quartiles of transformed values of VAS and TTO for each health state. Differences between the means for TTO and VAS are generally less than 0.1 (Table 3).

**Table 3:** Mean, standard deviation, median and quartiles for rescaled VAS and TTO values (*n* = 313)

States	VAS					TTO				
	Mean	Std. Deviation	P25	P50	P75	Mean	Std. Deviation	P25	P50	P75
11111	1.0000	0.0000	1	1	1	1.0000	0.0000	1	1	1
31122	0.6874	0.1628	0.6	0.7	0.8	0.6125	0.1685	0.5	0.6	0.7
22113	0.5679	0.1858	0.4	0.6	0.7	0.5193	0.1613	0.4	0.5	0.6
13212	0.6178	0.1797	0.5	0.6	0.7	0.4867	0.1632	0.4	0.5	0.6
12321	0.5882	0.1577	0.5	0.6	0.7	0.5025	0.1465	0.4	0.5	0.6
21231	0.5171	0.1377	0.4	0.5	0.6	0.4816	0.1383	0.4	0.5	0.5
22222	0.4613	0.1468	0.4	0.5	0.5	0.4155	0.1556	0.3	0.4	0.5
23311	0.4451	0.1387	0.4	0.4	0.5	0.4300	0.1483	0.4	0.4	0.5
11332	0.4300	0.1454	0.3	0.4	0.5	0.4503	0.1446	0.4	0.5	0.5
32131	0.3962	0.1422	0.3	0.4	0.5	0.3963	0.1486	0.3	0.4	0.5
31213	0.4962	0.1857	0.4	0.5	0.6	0.3980	0.1450	0.3	0.4	0.5
13123	0.4553	0.1307	0.4	0.5	0.6	0.4426	0.1431	0.4	0.5	0.5
12233	0.4335	0.1921	0.3	0.4	0.6	0.3842	0.1941	0.3	0.4	0.5
33221	0.4764	0.2063	0.3	0.4	0.6	0.3835	0.1412	0.3	0.4	0.4
21323	0.4006	0.1817	0.3	0.4	0.6	0.3647	0.1381	0.3	0.4	0.4
23132	0.3946	0.2201	0.2	0.4	0.5	0.2794	0.1550	0.2	0.2	0.3
32312	0.3884	0.2301	0.2	0.4	0.5	0.2904	0.1428	0.2	0.3	0.3
33333	−0.1378	0.2465	−0.2	−0.1	0	−0.0801	0.1509	−0.125	−0.1	−0.028
Total	0.4788	0.2703	0.3	0.5	0.6	0.4310	0.2457	0.3	0.4	0.5

### Regression analyses

Four models and the goodness-of-fit indices for each are shown in Table 4. All models and all coefficients were statistically significant (*P* < 0.05). All models passed the Breusch-Pagan/Cook-Weisberg test, which indicated the presence of homoscedasticity. Four regression coefficients for each dummy variable are very close to one another (Table 4). The greatest difference is only 0.059 produced by PD3 between Model 1 and Model 2; PD2 produces the least difference of 0.01 between Model 1 and Model 2.

**Table 4:** Coefficients and indices of the goodness-of-fit of VAS and TTO models

	Model 1 (VAS)	Model 2 (TTO)	Model 3 (VAS)	Model 4 (TTO)
11111	1	1	1	1
constant	− 0.0299	− 0.0141	–	–
MO2	− 0.1154	− 0.1292	− 0.1158	− 0.1340
MO3	−0.2180	− 0.2058	− 0.2111	−0.2159
SC2	−0.1060	− 0.1329	− 0.1064	−0.1377
SC3	−0.2172	−0.2220	− 0.2103	−0.2321
UA2	−0.0879	−0.1170	− 0.0884	−0.1218
UA3	−0.2413	−0.2103	− 0.2345	−0.2205
PD2	−0.0767	−0.0673	− 0.0772	−0.0721
PD3	−0.2553	−0.1967	− 0.2484	−0.2068
AD2	−0.0646	−0.1096	− 0.0650	−0.1145
AD3	−0.2087	−0.1893	− 0.2018	−0.1994
N3	0.0439	−0.0318	–	–
Adj. R-square	0.9409	0.9499	0.9550	0.9664
AIC	− 5285.48	− 6918.29	− 5252.47	− 6876.14
BIC	− 5192.57	− 6825.38	− 5172.83	− 6796.50
MAE	0.0304	0.0269	0.0327	0.0310
# > 0.05	4	1	4	4
# > 0.1	0	0	0	0
r	0.9847	0.9879	0.9813	0.9867
Logical error num.	0	0	0	0

All models and regression coefficients were significant (*P* < 0.05); *Adj. R-square* adjusted R-square, *AIC* Akaike information criterion, *BIC* Bayesian information criterion, *MAE* mean absolute error between observed mean and predicted value # > 0.05, number of MAE > 0.05 out of 18 states; # > 0.1, number of MAE > 0.1 out of 18 states, r,correlation coefficient between observed means and predicted values, Logical error num., number of inconsistencies among all predicted health state values

High levels of the goodness-of-fit statistics are seen for all models. Adjusted-R squares all exceed 0.94. Pearson correlation coefficients (r) between observed means and predicted values are higher than 0.98. Mean absolute error (MAE) between observed means and predicted values is less than 0.04. No errors in logic were observed among the predicted values of 243 health states.

**Fig. 1:**
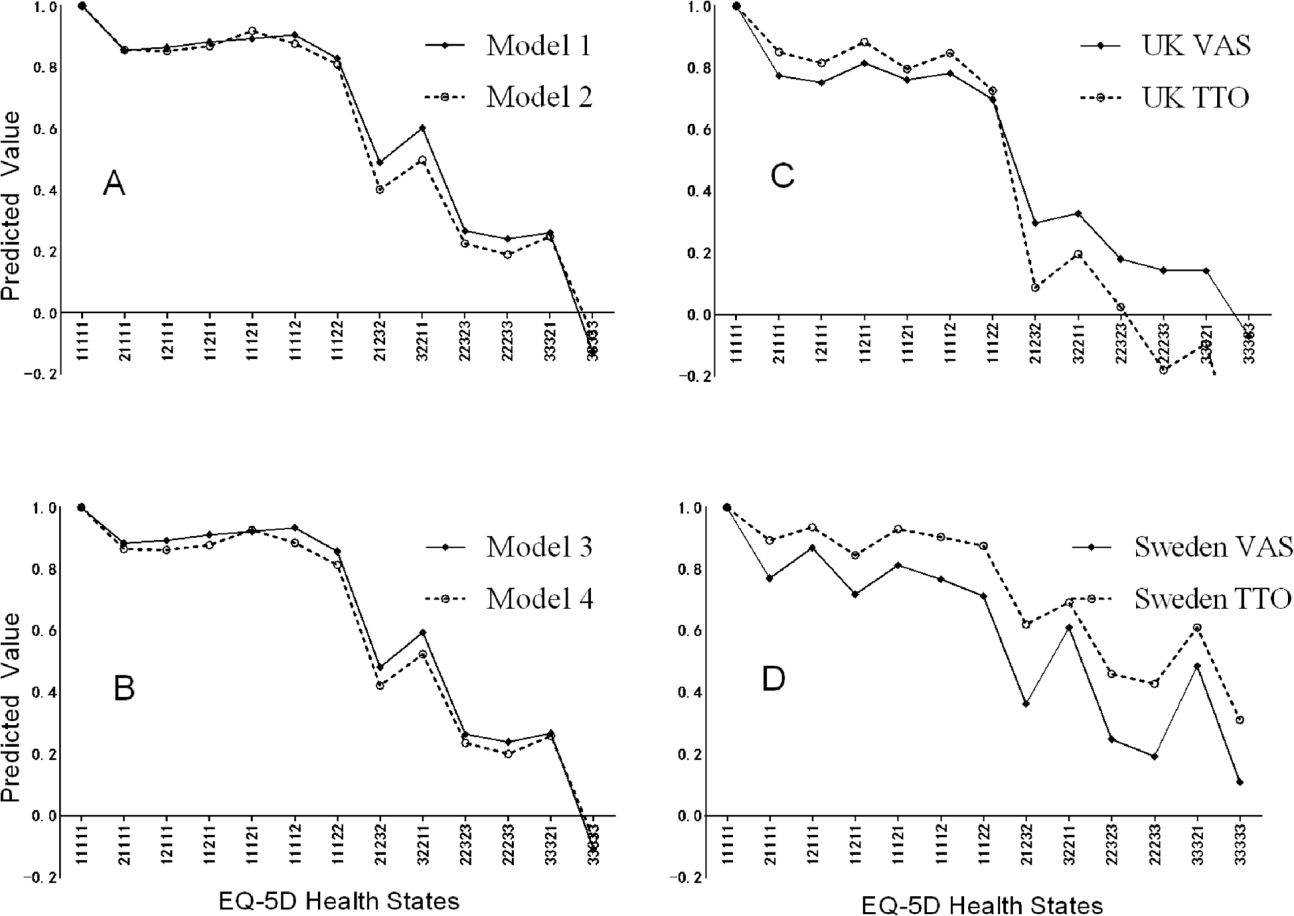
**a** Curves of Model 1 and Model 2 perform similarly, **b** Curves of Model 3 and Model 4 perform more similarly, **c** Curves of UK VAS and UK TTO cross over each other, **d** Curves of Sweden VAS is lower than Sweden TTO

Figure 1a delineates the predictions derived from Model 1 (VAS) and Model 2 (TTO). Figure 1b delineates the predictions derived from Model 3 (VAS) and Model 4 (TTO). The points of each health state in Fig. 1a and b are almost overlapping except the states of 21232 and 32211, which differ slightly. This indicates that similar results obtained under certain conditions supports different model specifications.

## Discussion

Previous studies comparing VAS and TTO have documented many defects regarding TTO, including that TTO generates more inconsistencies than VAS [46]; TTO is burdensome [31]; TTO values are negatively related to the duration of optimal health states and positively related to the poorer health states [24]; TTO is prone to interviewer effects [47]. After controlling some conditions through the design of the present study, we found that VAS and TTO generate similar responses. Comparing the predicted values in our study (Fig. 1a and b) to prior reports in the literature (Fig. 1c and d) [21, 22], results derived from the present are more similar. Four specific features of our study design provide a basic framework for explaining the discrepancies between VAS and TTO.

The key explanation for the ability to generate similar responses was the homogeneous sample chosen from a medical university. Medical students are superior to the general population as study subjects in two respects. First, they have less difficulty in understanding the TTO method than the general population. It was widely reported that TTO is more difficult to understand than VAS for the general population [31, 36, 48]. Medical students are young, enthusiastic, and committed to health and healthcare. Therefore, they are able to reduce error in the face of the complex TTO valuation task. Second, medical students are highly adherent. The TTO method involved a term of “immediate death”, which can elicit antipathy and non-adherence in the general population [49, 50]. Medical students are more comfortable with these concepts, more devoted to improving the quality of life of their patients, and thereby more likely to complete the trade-off task.

Adopting an adjusted scale is the second important contributor to the similar responses we demonstrated. The scale used in our study is not the same as the 20 cm vertical scale calibrated from 0 to 100 that is standard in EQ-VAS. We used a scale of − 10 ~ 0 ~ 10 for both methods for several reasons. First, we sought to apply the same scale in the elicitation, to minimize systematic measurement error. Second, by using revised scale, VAS possesses explicit anchor points as TTO does, and an anchored scale has the advantage of simplifying the process of the VAS valuation. Third, the range of − 10 ~ 0 ~ 10 has fixed boundaries for the transformed values of HRQoL. Fixed boundaries are critical in the process of elicitation and modeling [45]. However, in the MVH protocol, both ranges of VAS and TTO methods are transformed into -∞ ~ 0 ~ 1 [36], so the lower boundary is unbounded. As Seymour et al. demonstrated, the “ceiling” effect can be controlled by adjusting VAS and TTO ranges between − 1 and 1 [51]. EQ-VT protocol, the latest version of TTO elicitation, has also adopted − 1 ~ 0 ~ 1 [37]. Dolan has also introduced a fix-boundary rescaling method for WTD states. Although there was a typo in the alternative formula, the idea of “compensation” reflects the essence of trade-offs [36].

The third point worthy of mention is the computer-assisted personal-interviewing process. As reported by Ramos-Goni et al., interviewer effects were identified in many valuation studies [52]. In the CAPI process, a standardized procedure of valuation was embedded to facilitate self-administration, and should eliminate the potential interviewer bias [34, 35, 53, 54]. Furthermore, the CAPI process has been successful in other research ways: assisting and simplifying. Assisting means that inconsistent and irrational values would be fed back to the participants in real time to improve the reliability of the valuation. In addition, the use of computer-assisted personal-interviewing process reduces the need for the “warm-up” steps of ranking and pairwise comparison, which should be advantageous given the finding that ranking leads to a higher rate of inconsistency than VAS [55].

Selecting fully balanced health states represents the final key factor contributing to similar responses. According to the definition of the QALYs, the utility values of health states must lie on an interval scale anchored at 0 (death) and 1 (full health). Therefore, fully balanced states are expected to receive well-distributed utility values. A total of 18 health states selected via an orthogonal design have the considerable advantage of balanced distribution, which should represent all possible levels for each dimension. There is no contradiction between the balanced distribution of health states and a stabilized standard deviation of utility values. The stabilized standard deviation also plays an important role in the modeling algorithms as well as facilitating the comparability of the resultant value sets. Additionally, Sun et al. suggested that reasonable parity of health states should produce better results [56].

Although most predicted values were close to each other based on methods of VAS and TTO, the two states of 21,232 and 32,211 were slightly separated. This suggests that there may be other factors which were overlooked. For example, Augestad et al. pointed out that the attitudes toward death may influence the value sets [44]. The use of “death” is inevitable in the TTO method. This essential difference between the VAS and TTO is difficult to eliminate. Additionally, the process of comparing the current health state to “death” might cause “noise” since it is metaphysically unknown [44]. Badia et al. found that the VAS is more feasible and reliable than TTO in the Spanish population [57]. Taking our results into consideration, one could conclude that VAS should occupy a position of relative advantage over TTO in the general population, especially to older adults living in rural areas.

In summary, this study surfaced some previously neglected biases and provided experimental evidence that VAS and TTO can generate similar results under specific conditions. The similarity might shed light on the intrinsic equality of both methods. VAS would therefore seem to serve as a substitute for TTO, especially in a general population survey due to its relative ease and convenience. The major strength of this study was its experimental study design. The main weakness is the presence of separation over a small number of health states, suggesting unmeasured characteristics. Despite these encouraging results, many unanswered questions remain, such as the extent to which the new scale influenced the VAS valuation, and identification of the effect of each restrictive condition. Future work would tackle these issues.

## Conclusions

The data reported here have further strengthened our speculation that VAS and TTO methods in valuation of EQ-5D health states could be intrinsically equivalent. If confirmed, the VAS method is more applicable than TTO for health valuation in the general population due to its simplicity and superior feasibility.

## Supplementary information

**Additional file 1.** The interfaces of VAS and TTO in the CAPI software.

## Data Availability

Raw data may be made available upon reasonable request from the corresponding author.

## Abbreviations

BTD: Better than death
CAPI: Computer-Assisted Personal Interviewing
EQ-5D: EuroQol 5 dimensions
EQ-VT: EuroQol Group Valuation Technology
HRQoL: Health-related Quality of Life
HUI: The Health Utility Index
MAUI: Multi-attribute utility-based instruments
MAE: The mean absolute error
GLS: General least squares
MVH: Measurement and Valuation of Health
SF-36: The Short Form of 36 questions
SG: The Standard gamble
TTO: The time trade-off
VAS: The visual analogue scale
WTD: Worse than death
