# Supplementary material for: Similar responses to EQ-5D-3L by two elicitation methods: visual analogue scale and time trade-off
Source: BMC Med Res Methodol. 2020 May 14;20:118. doi: 10.1186/s12874-020-01008-9 (PMC7227357; doi:10.1186/s12874-020-01008-9)
Supplement: Supplementary file 1 — Additional file 1. The interfaces of VAS and TTO in the CAPI software. [file 12874_2020_1008_MOESM1_ESM.doc]

**Supplemental Material01**


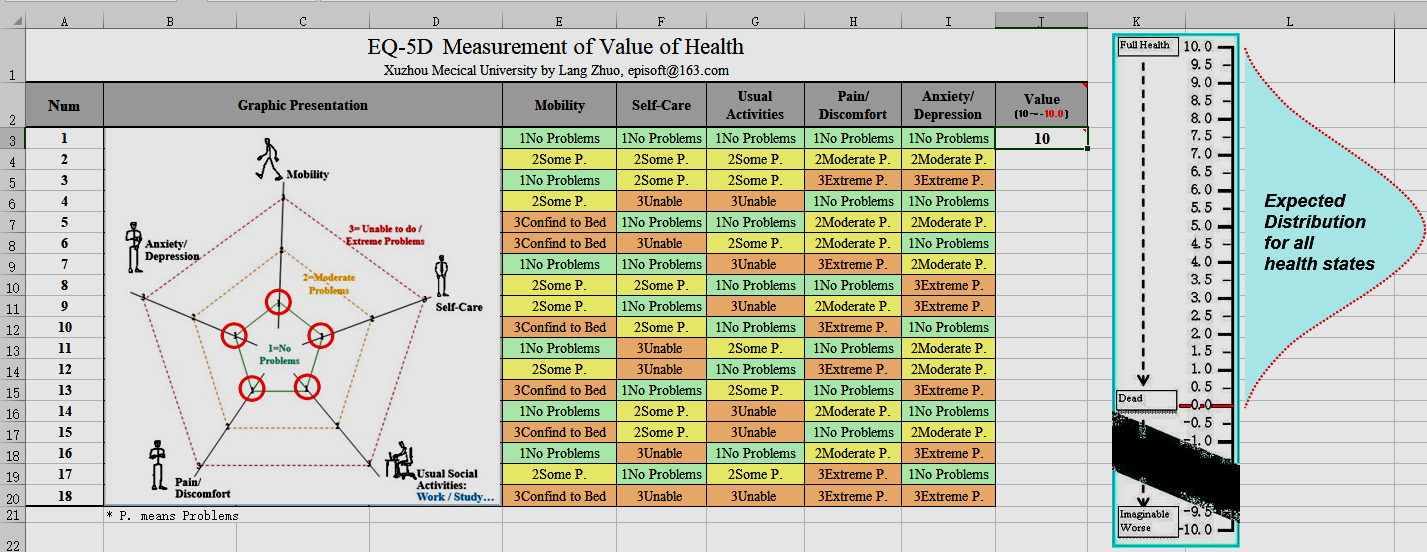


Figure S1 VAS interface of the CAPI software

**
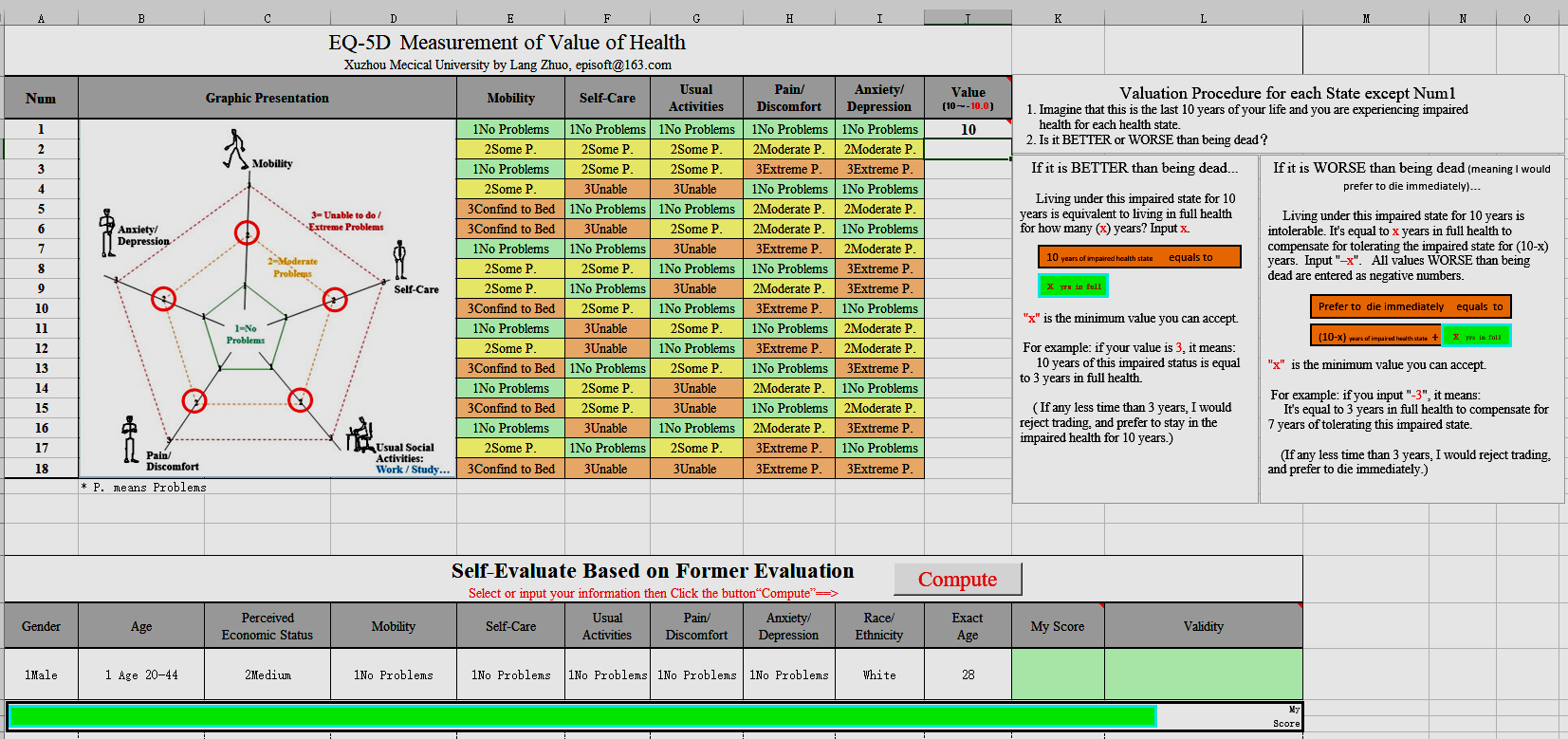
**

Figure S2 TTO interface of the CAPI software
